# Deficiency in Cardiolipin Reduces Doxorubicin-Induced Oxidative Stress and Mitochondrial Damage in Human B-Lymphocytes

**DOI:** 10.1371/journal.pone.0158376

**Published:** 2016-07-19

**Authors:** Baikuntha Aryal, V. Ashutosh Rao

**Affiliations:** Laboratory of Applied Biochemistry, Division of Biotechnology Review and Research III, Office of Biotechnology Products, Office of Pharmaceutical Quality, Center for Drug Evaluation and Research, U.S. Food and Drug Administration, Silver Spring, Maryland, 20993, United States of America; National Institutes of Health, UNITED STATES

## Abstract

Cardiolipin (CL) is an inner mitochondrial membrane phospholipid which plays an important role in mitochondrial function. Perturbation in CL biosynthesis alters mitochondrial bioenergetics causing a severe genetic disorder commonly known as Barth syndrome. Barth syndrome patients are known to have a reduced concentration and altered composition of CL. Cardiolipin is also known to have a high affinity for the chemotherapeutic agent doxorubicin (Dox), resulting in an extensive mitochondrial accumulation of the drug. Our results indicate that B-lymphocytes from healthy individuals are more sensitive to Dox-induced oxidative stress and cellular toxicity compared to the B-lymphocytes from Barth syndrome as indicated by greater cell death and greater level of cleaved caspase-3 following Dox treatment. Barth lymphocytes, when compared to healthy lymphocytes, showed a greater basal level of mitochondrial reactive oxygen species (mito-ROS), yet exhibited a lower level of induced mito-ROS production in response to Dox. Significantly less ATP content and slightly greater OXPHOS protein levels were detected in healthy cells compared to Barth cells after Dox treatment. Consistent with greater mitochondrial ROS, treatment with Dox induced a higher level of lipid peroxidation and protein carbonylation in healthy lymphocytes compared to Barth lymphocytes. The final remodeling of CL during CL synthesis is catalyzed by the tafazzin protein. Knockdown of *tafazzin* gene in H9c2 cardiomyocytes using siRNA showed decreased oxidant-induced damage, as observed in Barth lymphocytes. Our findings demonstrate that a deficiency in CL might provide a therapeutic advantage in favor of oxidant-induced anticancer activities.

## Introduction

Reactive oxygen species (ROS) are byproducts of the metabolic process. Physiological production of ROS is critical for cell signaling and homeostasis, and the concentration of ROS is balanced by a complex cellular antioxidant system under normal conditions [1]. Excessive production of ROS in response to various pathological conditions and therapeutic drugs causes oxidative damage to lipids, nucleic acids, and proteins causing cell death. Mitochondria are the major site of ROS production due to perturbation in mitochondrial oxidative phosphorylation (OXPHOS) under normal or drug-induced toxic conditions [2]. Cardiolipin (CL) is an inner mitochondrial membrane specific phospholipid that plays a critical role in maintaining mitochondrial bioenergetics and mitochondrial membrane potential [3]. Mitochondrial CL contains three glycerol backbones and four acyl chains resulting in a specific conical ultrastructure distinct from other phospholipids. In mitochondria, CL is associated with maintaining proper function of the respiratory chain protein complexes [4]. A deficiency of CL destabilizes the structural integrity of mitochondrial protein complexes causing electron leakage and excessive ROS production leading to oxidative damage to nucleic acid and proteins [3,5,6].

Barth syndrome is an X-linked recessive disease characterized by cardiac and skeletal myopathy, neutropenia, and growth retardation. Barth Syndrome is caused by the mutations in the *tafazzin* gene located on chromosome Xq28 [7]. Tafazzin is a phospholipid acyltransferase that catalyzes the remodeling of CL at the final stage of biosynthesis [8]. Mutations in tafazzin cause a decrease in tetra-linoleoyl specific CL and accumulation of monolysocardiolipin species within the inner mitochondrial membrane. Barth syndrome patients exhibit a reduced concentration and altered composition of CL in the heart, lymphocytes, fibroblasts, and skeletal muscles [9,10]. A decrease in CL content has been associated with aging, affecting the OXPHOS system in mitochondria [11,12]. Alternations of CL have also been reported under various pathological conditions including traumatic brain injury, heart failure, ischemia-reperfusion injury, muscles weakness, neurodegenerative diseases, diabetes and cancer cachexia [13,14].

Doxorubicin (Dox) is a potent anticancer drug but its clinical application has been limited due to its dose-dependent adverse side effects including cardiomyopathy and heart failure. One of the widely accepted mechanisms of Dox-induced cardiotoxicity is the generation of excessive reactive oxygen species (ROS) through iron-mediated redox cycling and oxidative damage to protein and nucleic acids leading to mitochondrial and bioenergetic failure and cell death by apoptosis [2]. The majority of Dox taken up by cells accumulates in the nucleus, yet a significant amount of Dox is also known to accumulate in the mitochondria [15]. CL is considered to play a critical role in the mitochondrial accumulation of Dox due to the formation of strong complexes with both Dox and Dox-Fe^3+^ complex [16–19]. Dox metabolites that accumulate in the inner mitochondrial membrane are easily reduced by complex I of the electron transport chain (ETC), producing excessive ROS and causing oxidative damage to ETC complexes. Thus, Dox-induced mitochondrial toxicity is, at least in part, due to the formation of a strong Dox-CL complex resulting in the retention of Dox in the inner mitochondrial membrane, permitting it to undergo continued but futile redox cycling and leading to extensive oxidative damage to mitochondria.

We hypothesize that a deficiency in mitochondrial CL content reduces Dox accumulation in mitochondria, thereby limiting its oxidative damage to mitochondria. In this paper, we used CL deficient B-lymphocytes derived from Barth patients and B-lymphocytes from healthy subjects to test our hypothesis. We selected EBV-transformed B-lymphocytes in our study because they represent an important tissue source of genetic information from patients of various diseases, and B- lymphocytes from Barth patients are known to have a deficiency in CL content [10,20].

## Materials and Methods

### Cell Culture

EBV-transformed B-lymphocytes derived from multiple, anonymous donors with Barth syndrome and from healthy volunteers were purchased from Coriell Cell Repositories (Camden, NJ) and cultured according to Coriell’s suggested protocol in RPMI 1640 supplemented with 2 mM L-glutamine, and 15% fetal bovine serum. Cells were maintained under standard cell culture conditions (37°C, 5% CO_2_, and 95% relative humidity) in an upright standing position. B-lymphocytes were treated with the relevant drug solutions in DMSO or DMSO alone (control) for indicated time points. The use of banked and anonymous human lymphocytes for research purposes was approved by the FDA’s Institutional Review Board for Research Involving Human Subjects (RIHSC). H9c2 cardiomyocyte cells were purchased from ATCC (Manassas, VA) and cultured in DMEM containing 10% FBS, 4 mM L-glutamine, and 1 mM sodium pyruvate.

### Isolation of Mitochondria

Mitochondria enriched fractions were prepared using previously published protocols with slight modifications [21–23]. Briefly, cells were isolated by centrifugation and washed twice with PBS at 4°C. Cells were resuspended in ice-cold hypotonic buffer (10 mM Tris-HCl, pH 7.6) containing protease and phosphatase inhibitor cocktails. The cell suspension was homogenized on ice using a glass/Teflon homogenizer. The number of strokes was optimized by inspecting homogenate under the microscope for intact cells. The cell homogenate was then gently passed five times through a 26G 1/2” needle using a 1 mL syringe and centrifuged at 1000 g for 10 minutes at 4°C in a 1.5 mL microcentrifuge tube. The supernatant was collected in a clean tube and centrifuged again at 1000 g for 10 minutes at 4°C. The supernatant containing the crude mitochondrial and cytosolic fractions was transferred to a new clean tube and a 1.5 mM sucrose solution was added to a final concentration of 180 mM. The supernatant containing mitochondrial and cytosolic fractions was then centrifuged at 14,000 g for 10 minutes to separate mitochondrial (pellet) and cytosolic fractions (supernatant). The pellet was resuspended in 500 μl of hypotonic solution containing a protease and phosphatase inhibitor cocktail before being centrifuged at 14,000 g for 10 minutes. The supernatant was discarded and the pellet (mitochondrial fraction) was washed once more with hypotonic buffer.

### Knockdown of Tafazzin

H9c2 cells were seeded in tissue culture flasks using DMEM growth medium without antibiotics. After overnight incubation at 37°C, 5% CO_2_, and 95% RH, cells were transfected for 48 h with 20 nM of ON-TARGETplus SMARTPOOL *tafazzin* siRNA or ON-TARGETplus non-coding (NTP) siRNA (Thermo Scientific, Waltham, MA). Following transfection, cells were treated with drugs for indicated time-points in fresh medium. After drug treatment, cells were either processed for ROS measurement by FACS analysis after staining with MitoSOX red, or lysed in cell lysis buffer to be analyzed by western blot.

### Measurement of cell death using annexin V-FITC/ propidium iodide

Cells were treated with 100 nM, 500 nM, and 1μM of Dox (Sigma Aldrich, St. Louis, MO) or 10 μM VP-16 for 24 h. After incubation with drugs, cells were harvested by centrifugation, washed twice with PBS, and stained using the Annexin V-FITC Apoptosis Detection Kit II (BD Pharmingen, San Jose, CA) containing Annexin V and propidium iodide as recommended by the manufacturer. Stained cells were analyzed by a FACSCalibur flow cytometer (BD Biosciences, San Jose, CA).

### Western blot analysis

Cells were treated with vehicle (DMSO) or indicated concentration of Dox for 24 h. After drug treatment, cells were harvested by centrifugation, washed with PBS, and lysed in whole cell lysis buffer (20 mM Tris-HCl pH 7.5, 150 mM NaCl, 1 mM EGTA, 1% Triton X-100, 1 mM Na_3_VO_4_) containing a protease and phosphatase inhibitor cocktail on ice. After centrifugation for 20 minutes at 16,000 g, supernatant was collected and total protein concentration in cell lysate was determined by BCA protein assay kit (Thermo Scientific, Rockford, IL). Following SDS-PAGE separation on a 4–12% bis-tris gel, protein was transferred onto PVDF membranes. Membranes were blocked for 1 h in blocking buffer (LI-COR, Lincoln, NE) followed by incubation in primary antibodies; cleaved caspase-3 (Cell Signaling Technology, Beverly, MA), LC3-II (Novus Biologicals, Littleton, CO), tafazzin (BosterBio, Pleasanton, CA), tubulin (Cell Signaling Technology, Beverly, MA), VDAC (EMD Millipore, Billerica, MA), or OXPHOS (Abcam, Cambridge, MA) for 3 h in a blocking buffer containing 0.1% Tween 20. After washing three times in washing buffer (1xPBS + 0.1% Tween 20), blots were incubated with IRDye 800CW secondary antibody (LI-COR, Lincoln, NE) for 1 h, washed three times with washing buffer, and the proteins of interest were detected and quantified using the Odyssey infrared imaging system (LI-COR, Lincoln, NE).

### Measurement of mitochondrial superoxide using MitoSOX Red

Mitochondrial superoxide was measured as described previously [24,25]. Briefly, cells were treated with indicated concentrations of Dox for 24 h followed by incubation with 2 μM MitoSOX Red for 30 minutes. After treatment, cells were washed three times with PBS buffer containing calcium and magnesium ions. The basal and drug-induced levels of mitochondrial superoxide were determined by measuring the mean fluorescence intensity of MitoSOX Red using the FACSCaliber Flow Cytometer (BD Bioscience, San Jose, CA). All mean fluorescence intensity values for MitoSOX Red were corrected for background fluorescence from the corresponding concentration of Dox fluorescence alone. The data are presented in the histogram as a fold change in the mean fluorescence intensity of MitoSOX Red of Dox treated sample normalized to control.

### Measurement of mitochondrial membrane potential (ΔΨ_m_)

Cells were treated with the indicated concentration of Dox for 4 h. Following Dox treatment, cells were incubated with pre-warmed solution of lipophilic cationic probe 5,5’,6,6’-tetrachloro-1,1’,3,3’-tetraethylbenzimidazolcarbocyanine iodide (JC-1) (ThermoFisher Scientific, Grand Island, NY) in growth medium at a concentration of 2 μg/mL for 30 minutes, washed with PBS, and assayed using a fluorescence plate reader (Molecular Devices, Sunnyvale, CA) with the following settings: excitation at 485 nm, emission at 540 nm (monomeric), and 590 nm (aggregates). After subtraction of background fluorescence for the wells containing cells without JC-1 dye, the ΔΨ_m_ was calculated using the ratio of the fluorescence intensity of 590 nm to 540 nm [26].

### Measurement of lipid peroxidation products

Cells were treated with Dox for 24 h and harvested by centrifugation. Lipid peroxidation product was assessed for control and Dox treated samples using a lipid peroxidation (MDA) colorimetric/Fluorometric assay following instructions from the manufacturer (BioVision, Milpitas, CA). The commercially purchased detection method is based on the reaction of MDA present in the test samples with thiobarbituric acid (TBA) to generate a colored MDA-TBA adduct which can be quantitatively measured by spectrophotometer at 532 nm. Amount of MDA for each treatment was normalized by protein concentration and expressed as nmol of MDA per mg of protein lysate. For comparison between healthy and Barth cells, amount of MDA for each treatment was normalized to amount of MDA calculated in control samples.

### Measurement of ATP level

Cellular ATP content was measured for control and Dox treated cells using a kit (Molecular Probes, Grand Island, NY). Healthy and Barth cells were treated with 0.5, 1, and 5 μM of Dox for 8 and 24 h, and ATP level was measured by Molecular Probes ATP determination kit following instructions from manufacturer. The amount of ATP was determined for each sample using an ATP calibration curve and expressed as pmol of ATP normalized by the number of cells. For comparison between healthy and Barth cells, amount of ATP for each treatment was normalized to control.

### Statistical analysis

Statistical analyses were performed using the software GraphPad Prism 6 (GraphPad Software, Inc., La Jolla, CA). ANOVA multiple comparision was used to analyze the significance of the results. Significant differences in Dox-induced changes between healthy and Barth lymphocytes or Taz siRNA and NTP siRNA transfected H9c2 cells treated with same concentration of Dox are provided in the figures. All values are expressed as mean ± SEM.

## Results

### Dox-induced apoptotic cell death is greater in healthy lymphocytes compared to Barth lymphocytes

**Fig 1:**
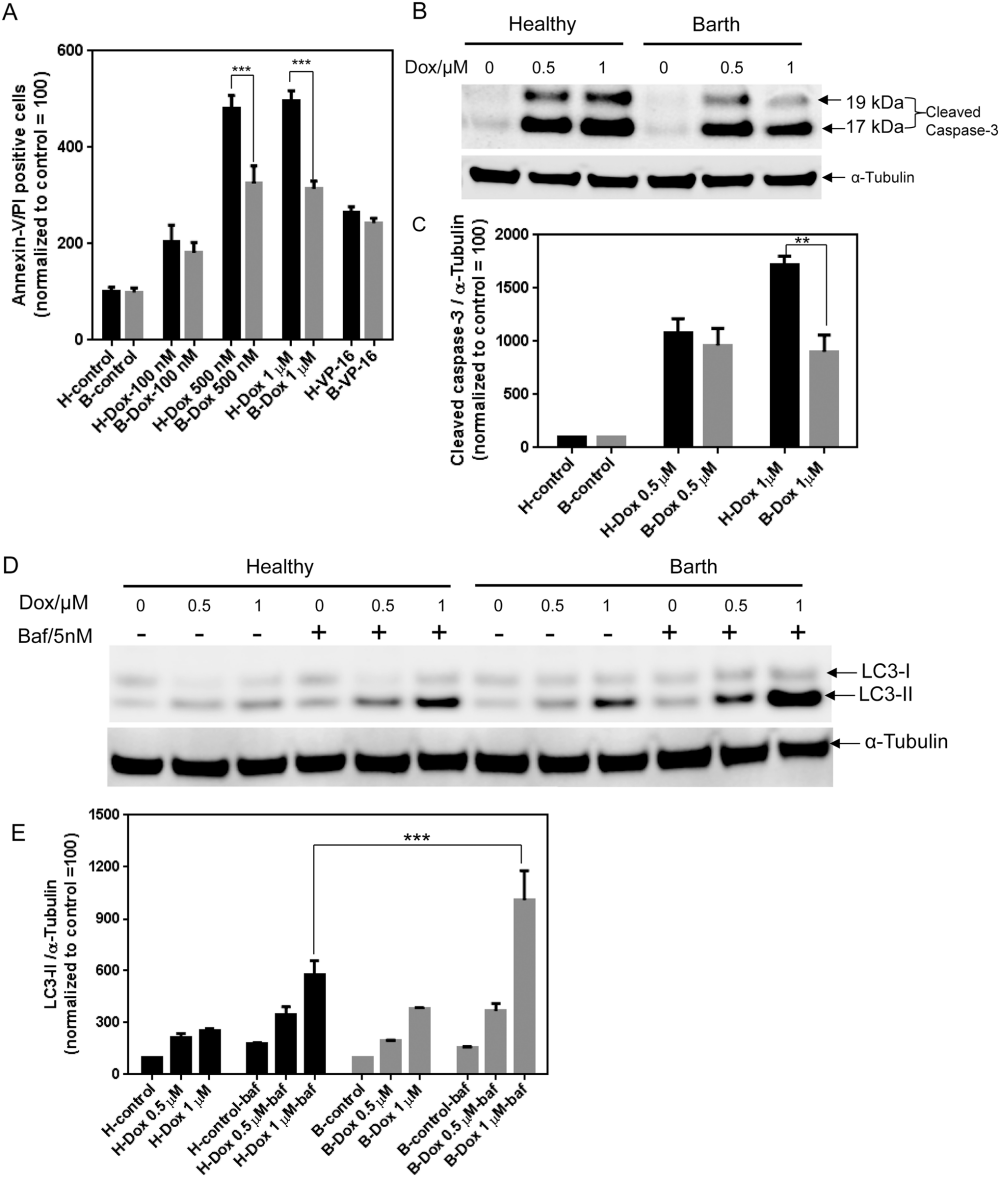
Dox-induced cell death in Barth and healthy lymphocytes. (A) Annexin V and propidium iodide staining was used to measure induction of apoptosis by 0.1, 0.5, and 1 μM of Dox following 24 h treatment. VP16 (10 μM) was used as a positive control. (B) Representative western blot showing the level of cleaved caspase-3 in healthy and Barth lymphocytes after 0.5 and 1 μM Dox treatment for 24 h. (C) Quantitative analysis of cleaved caspase-3 from (B) normalized by α-tubulin. Cleaved caspase-3 levels for control (untreated) healthy and Barth lymphocytes were normalized to 100 and Dox-induced changes in cleaved caspase-3 were normalized against corresponding control. (D) Representative western blot showing the level of autophagy induction determined by LC3-II levels in healthy and Barth lymphocytes after 0.5 and 1 μM Dox treatment for 24 h with or without 5 nM bafilomycin A1 for final 2 h of Dox treatment. (E) Quantitative analysis of LC3-II from (D) normalized by α-tubulin. LC3-II levels for control (untreated) healthy and Barth lymphocytes were normalized to 100 and Dox-induced changes in LC3-II level in the presence or absence of bafilomycin A1 were normalized against corresponding control. *p<0.05; **p<0.001; H = healthy B-lymphocytes, B = Barth B-lymphocytes, Baf = bafilomycin A1.

To investigate whether cardiolipin deficiency protects cells against doxorubicin-induced apoptosis, we compared Barth and healthy lymphocytes in the presence or absence of increasing concentrations of Dox. Dox-induced apoptosis was assessed by FACS analysis using Annexin V–PI staining (Fig 1A). Compared to DMSO (vehicle) treated control cells, a significant increase in Annexin V-PI positive cells were observed after Dox treatment for 24 h. This increase was higher in healthy lymphocytes compared to Barth lymphocytes. At a low Dox concentration of 100 nM, there were low levels of apoptotic cells and no significant difference in Annexin V-PI positive cells between healthy and Barth but at 500 nM or higher Dox concentration there was a significant increase in Annexin V-PI positive healthy cells compared to Barth cells. This provided the basis for testing 500 nM or higher concentration of Dox in the rest of our experiments.

Dox-induced apoptosis was confirmed by monitoring the cleaved caspase-3 level in healthy and Barth lymphocytes using western blot analysis. We observed two bands corresponding to the cleaved products of caspase-3 at 17 kDa and 19 kDa (Fig 1B). The cleaved product of caspase-3 at 17 kDa and partially cleaved product at 19 kDa have previously been reported in different cells [27]. Western blot analysis showed a higher level of Dox-induced cleaved caspase-3 in healthy lymphocytes compared to Barth lymphocytes (Fig 1B and 1C). These results confirm that healthy cells are more sensitive to Dox-induced apoptosis.

Autophagy is considered a catabolic process and a cell survival mechanism, but it can also lead to cell death if the excessive damage is not repaired. To determine whether autophagy can modulate Dox-induced cell death in B-lymphocytes, we analyzed the common autophagy related protein LC3-II in Dox-treated healthy and Barth cells. LC3-II was determined with or without addition of bafilomycin A1, a lysosomal acidification inhibitor, to test the occurrence of autophagy flux. α-Tubulin was used a loading control to quantify the levels of LC3-II before and after treatment [28,29]. The levels of LC3-II were increased after Dox and bafilomycin A1 treatment but this increase was greater in Barth lymphocytes compared to healthy lymphocytes (Fig 1D and 1E). Given that autophagy is a protective mechanism against cell death, an increase in LC3-II signal indicates that Barth cells are more resistant to Dox-induced cell killing at lower concentration. This data is also in agreement with the increased cell killing observed in healthy cells in panel A and B. It was noted that at higher concentrations of Dox (5 μM and above), we observed a decrease in autophagy in both cell lines (data not shown), indicating that autophagy may not be capable of protecting cells beyond a certain threshold of Dox concentration.

Overall, we confirmed that the deficiency of cardiolipin resulted in decreased apoptosis and increased autophagy under cytotoxic conditions.

### Mitochondrial accumulation of Dox is higher in healthy lymphocytes

**Fig 2:**
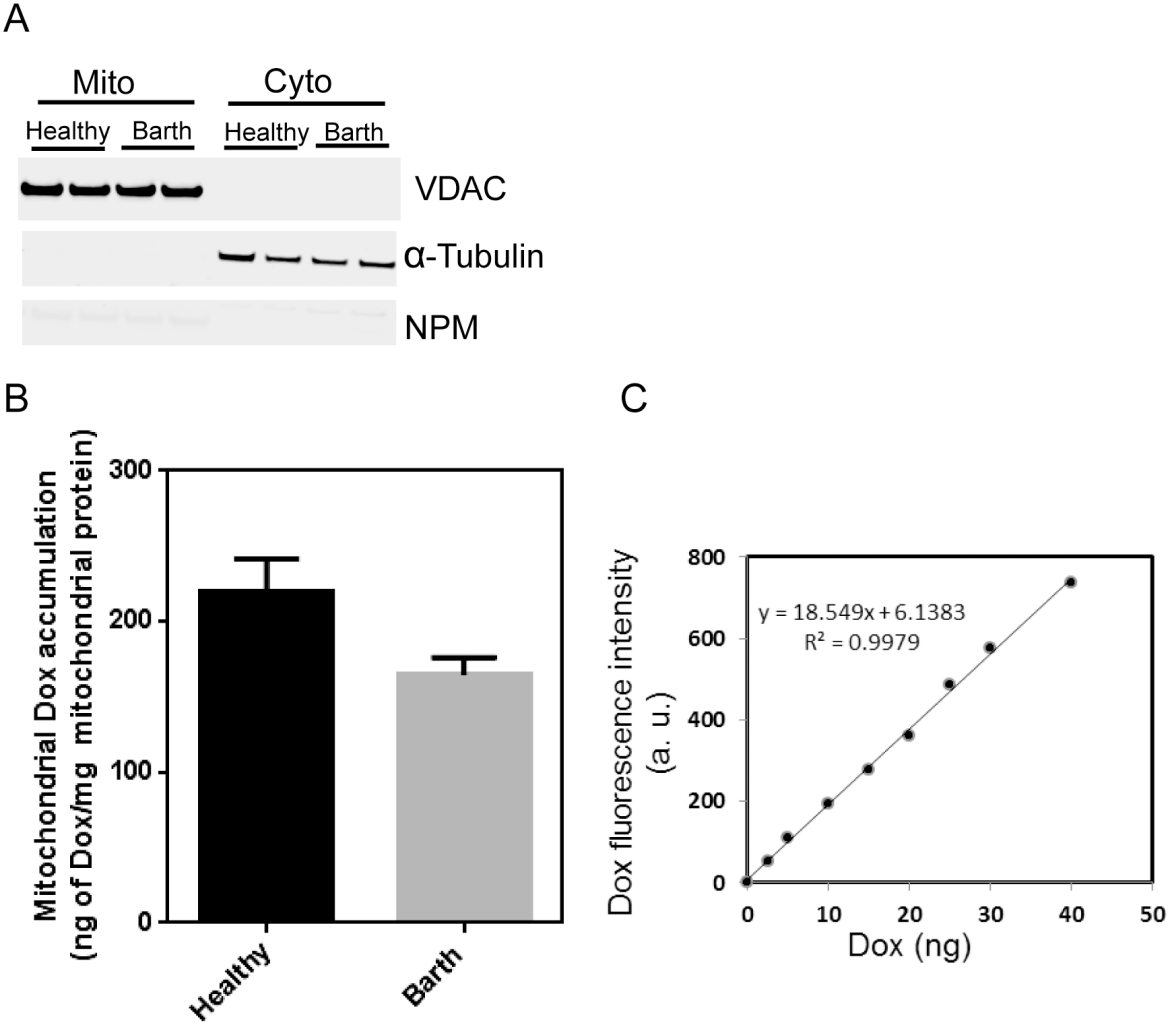
Mitochondrial accumulation of Dox in healthy and Barth lymphocytes. (A) Representative western blot showing the purity of the mitochondrial fractions. Protein lysates from cell fractionation were loaded in duplicate for healthy and Barth lymphocytes. (B) Quantitative determination of mitochondrial Dox by fluorescence method (λ_ex_ = 478 nm λ_em_ = 594 nm) in healthy and Barth cells. Cells were incubated with 1μM Dox for 24 h, washed with PBS, then incubated in fresh media without Dox for 2 h, then cells were harvested and subjected to isolate the mitochondrial fractionation for Dox measurement. (C) Standard curve generated from the known concentrations of Dox solutions that was used to quantify mitochondrial Dox.

To test whether the difference in cytotoxicity between Barth and healthy cells was due to differences in mitochondrial drug uptake, we tested mitochondrial drug accumulation in both cells. Following isolation of the mitochondrial fraction from the Dox treated healthy and Barth lymphocytes, the amount of Dox in the mitochondrial fraction was determined by measuring Dox fluorescence λ_ex_ at 478 nm and λ_em_ at 594 nm [30]. Quantification of Dox was achieved using a standard curve generated from known concentrations of Dox. The mitochondrial fraction from healthy lymphocytes treated with Dox showed a higher Dox fluorescence compared to the mitochondrial fraction from Barth lymphocytes (Fig 2B). This indicates that Dox accumulates to a greater extent in healthy cell mitochondria compared to the mitochondria from CL deficient Barth cells.

### Dox-induced mitochondrial superoxide production is higher in healthy lymphocytes compared to Barth lymphocytes

**Fig 3:**
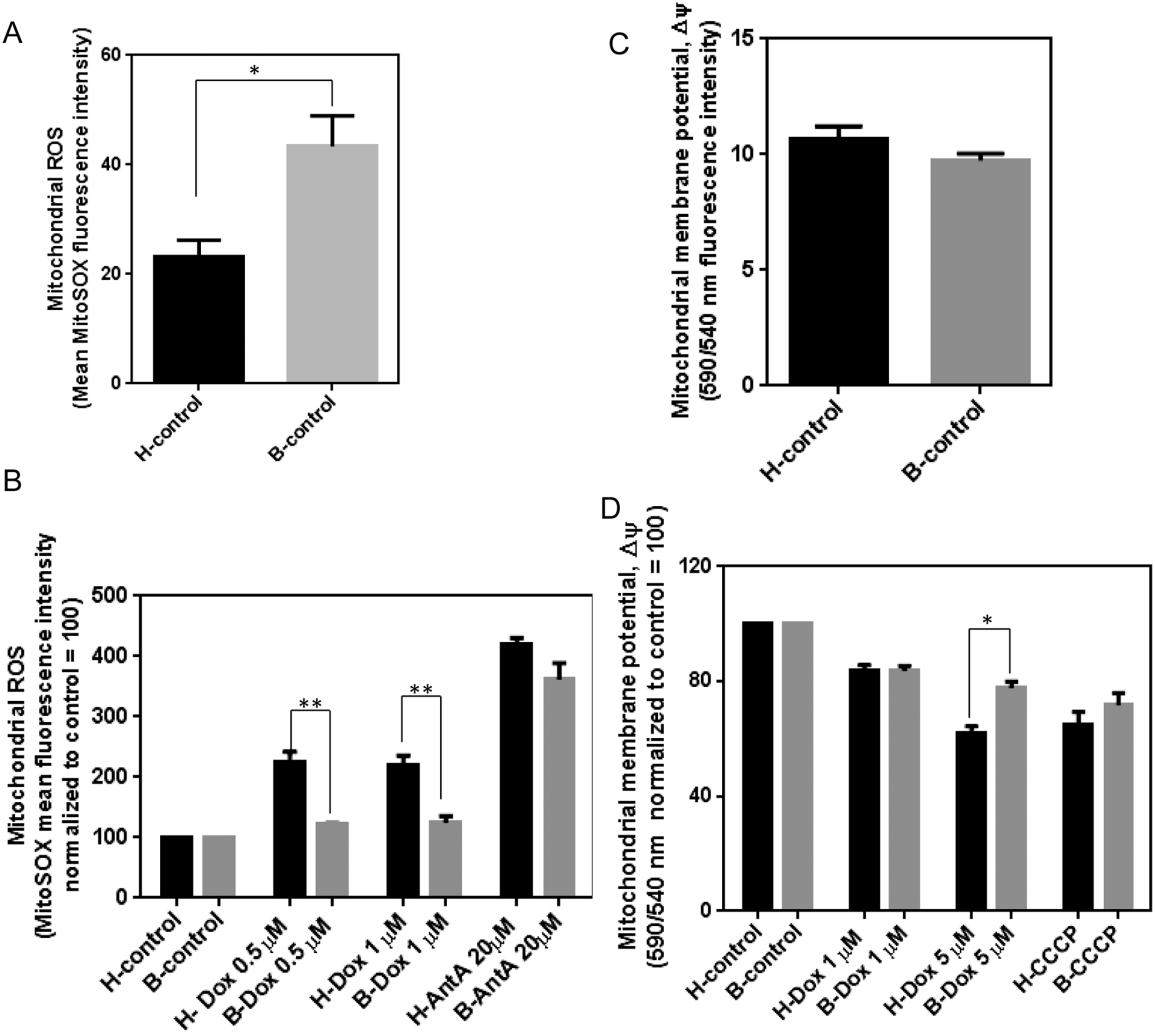
Determination of Dox-induced mitochondrial superoxide production and mitochondrial membrane potential in healthy and Barth lymphocytes. Quantitative analysis of changes in mean fluorescence intensity of mitochondrial superoxide sensitive dye MitoSOX Red (A) in healthy and Barth lymphocytes at basal level without Dox treatment and (B) after 0.5 and 1 μM Dox treatment for 24 h. Mitochondrial complex III inhibitor Antimycin A (20 μM) was used as a positive control. All values were normalized to mean fluorescence intensity of control. Quantitative representation of mitochondrial membrane potential of healthy and Barth lymphocytes at basal levels without Dox treatment (C), and after treatment with 1 and 5 μM Dox (D). CCCP (10 μM), a commonly used mitochondrial membrane depolarization agent was used as a positive control. To determine the Dox-induced oxidative stress between healthy and Barth lymphocytes, basal levels of ROS for control (untreated) healthy and Barth lymphocytes were normalized to 100; and Dox-induced changes in the ROS were normalized against corresponding control. Mitochondrial membrane potential was analyzed in a similar way. *p<0.05; **p<0.001; AntA = Antimycin A.

MitoSOX Red fluorescence dye is used to determine mitochondrial superoxide level by measuring mean fluorescence intensity using flow cytometry and this technique has been validated with fluorescence microscopy [24,25]. We hypothesized the decreased mitochondrial accumulation of Dox in Barth cells might result in decreased mitochondrial ROS. To understand the Dox-induced superoxide production in mitochondria and mitochondrial oxidative damage, we treated healthy and Barth lymphocytes with Dox for 24 h followed by 2 μM MitoSOX red for 30 minutes. Antimycin A, an inhibitor of ETC complex III, served as a positive control for mitochondrial superoxide generation. Our data shows that Dox-induced mitochondrial superoxide production is greater in healthy lymphocytes compared to Barth lymphocytes while the basal level of superoxide is greater in Barth lymphocytes compared to healthy lymphocytes (Fig 3A and 3B). Overall, our results indicate that mitochondrial retention of Dox is greater in healthy cells compared to Barth cells and CL deficient cells are better protected from Dox-induced oxidative damage compared to normal healthy cells.

We then tested the Dox-induced disruption of the mitochondrial membrane by determining the mitochondrial membrane potential in healthy and Barth lymphocytes following Dox treatment. Potential-dependent accumulation of JC-1 dye have been well studied in mitochondria, indicated by a shift in fluorescence emission from green (540 nm) to red (590 nm). Thus mitochondrial depolarization is determined by measuring a decrease in 590/540 nm fluorescence intensity ratio. Carbonyl cyanide m-chloro phenyl hydrazine (CCCP), a commonly used potent mitochondrial oxidative phosphorylation uncoupler served as a positive control. At basal levels, Barth lymphocytes have a reduced mitochondrial membrane potential compared to healthy cells (Fig 3C), in agreement with the increased basal superoxide found in panel A. Exposure of cells to Dox (1 and 5 μM) for 4 h led to membrane depolarization in both cell types, indicated by a decrease in the 590/540 nm fluorescence ratio, yet this depolarization was higher in healthy lymphocytes compared to Barth lymphocytes (Fig 3D). This difference in loss of mitochondrial membrane potential between healthy and Barth lymphocytes demonstrates greater Dox-induced mitochondrial damage in healthy lymphocytes compared to Bath lymphocytes.

### Dox-induced lipid peroxidation and protein carbonylation is higher in healthy lymphocytes compared to Barth lymphocytes

**Fig 4:**
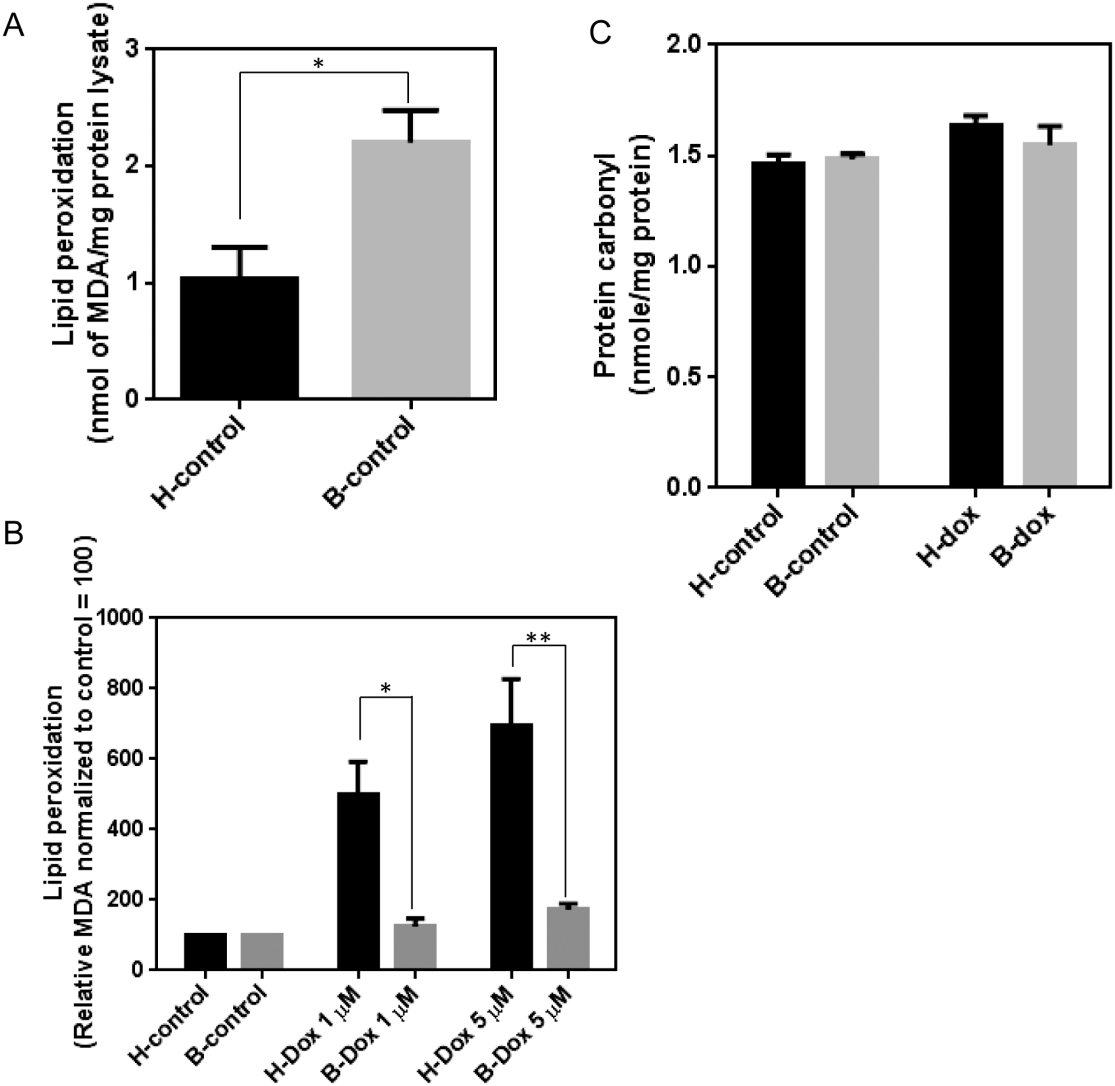
Determination of lipid peroxidation and total protein carbonylation in healthy and Barth lymphocytes. Quantitative determination of lipid peroxidation product in (A) healthy and Barth lymphocytes at basal level and (B) Dox-induced lipid peroxidation in healthy and Barth lymphocytes normalized to control. MDA-TBA adduct was measured at 532 nm and normalized to mg of protein in the cell lysate. To determine the Dox-induced changes in lipid peroxidation between healthy and Barth lymphocytes, basal levels of MDA for control (untreated) healthy and Barth lymphocytes were normalized to 100; and Dox-induced changes in MDA level were normalized against corresponding control. (C) Total protein carbonylation in healthy and Barth lymphocytes with and without Dox (1μM) treatment was determined by ELISA. *p<0.05; **p<0.001.

To understand the consequences of differential Dox-induced oxidative stress in healthy and Barth cells, we measured the lipid peroxidation product in the cell lysates. Among several lipid peroxidation products, malondialdehyde (MDA) has been extensively studied and is one of the most abundant aldehydes formed from the peroxidation of omega-3 and omega-6 fatty acids [31]. MDA has been used as a marker of lipid peroxidation because of its reaction with thiobarbituric acid (TBA) to yield a colored fluorescent adduct [32]. The formation of MDA-TBA adduct was quantified colorimetrically (OD = 532 nm). At basal conditions, MDA level was higher in Barth lymphocytes compared to healthy (Fig 4A). This is consistent with greater basal levels of ROS in Barth lymphocytes compared to healthy cells. After Dox treatment, there was an increase in lipid peroxidation product in both cell types, but this increase was significantly greater in healthy lymphocytes compared to Barth lymphocytes (Fig 4B). Our data indicates that Dox-induced oxidative damage is more extensive in healthy cells compared to CL deficient Barth cells.

To further understand the consequences of oxidative stress, we measured total protein carbonylation in Dox-treated healthy and Barth lymphocytes using ELISA. There was an increase in total protein carbonylation in both healthy and Barth lymphocytes after Dox treatment but overall Dox-induced total protein carbonylation was slightly greater in healthy lymphocytes compared to Barth lymphocytes (Fig 4C), although not statistically significant.

### Dox reduces ATP production to a greater extent in healthy lymphocytes compared to Barth lymphocytes

**Fig 5:**
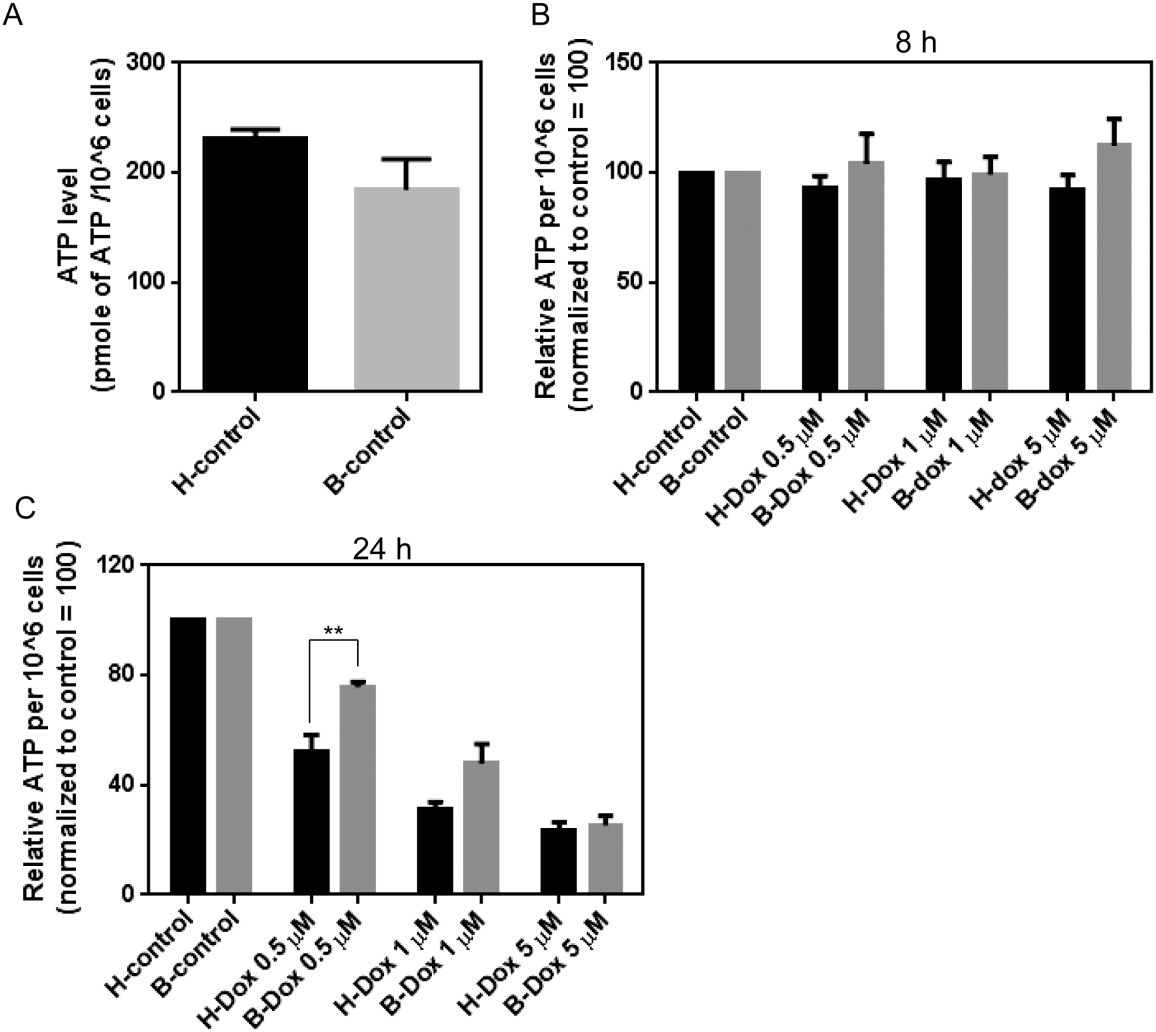
Determination of Dox-induced ATP levels in healthy and Barth lymphocytes. Healthy and Barth lymphocytes were treated with vehicle (control) or 0.5, 1, and 5 μM of Dox for 8 and 24 h and cellular ATP content was determined (A) at basal levels without Dox treatment and (B-C) in control and Dox-treated lymphocytes using a kit from Molecular Probes. Amount of ATP was calculated as pmol of ATP per 1x10^6^ cells. The y-axis shows the amount of ATP per 1x10^6^ cells. To determine the Dox-induced changes in the ATP level between healthy and Barth lymphocytes, basal level of ATP for control (untreated) healthy and Barth lymphocytes were normalized to 100; and Dox-induced changes in ATP level were normalized against corresponding control. **p<0.001.

Exposure to Dox is known to alter the ATP production in cultured cancer cell lines and cardiac tissue [33,34]. To confirm the effect of Dox on mitochondrial energy metabolism and to determine if there is any difference in ATP content between healthy and Barth lymphocytes following Dox treatment, we measured ATP levels after Dox treatment. After 8 h of Dox treatment there was no difference in ATP level between control and Dox treated cells, but at 24 h a significant decrease in ATP level was observed in Dox treated cells compared to control (Fig 5B and 5C). Dox treatment resulted in a significantly greater reduction in ATP level in healthy compared to Barth lymphocytes. This reinforces that Dox-induced mitochondrial oxidative damage, manifested as ATP production, is greater in healthy cells compared to Barth cells. As we observed previously, high concentrations of Dox at 5 mM had a plateaued effect that did not differ between the cell lines.

### Dox-induced increases in OXPHOS protein levels are higher in healthy lymphocytes compared to Barth lymphocytes

**Fig 6:**
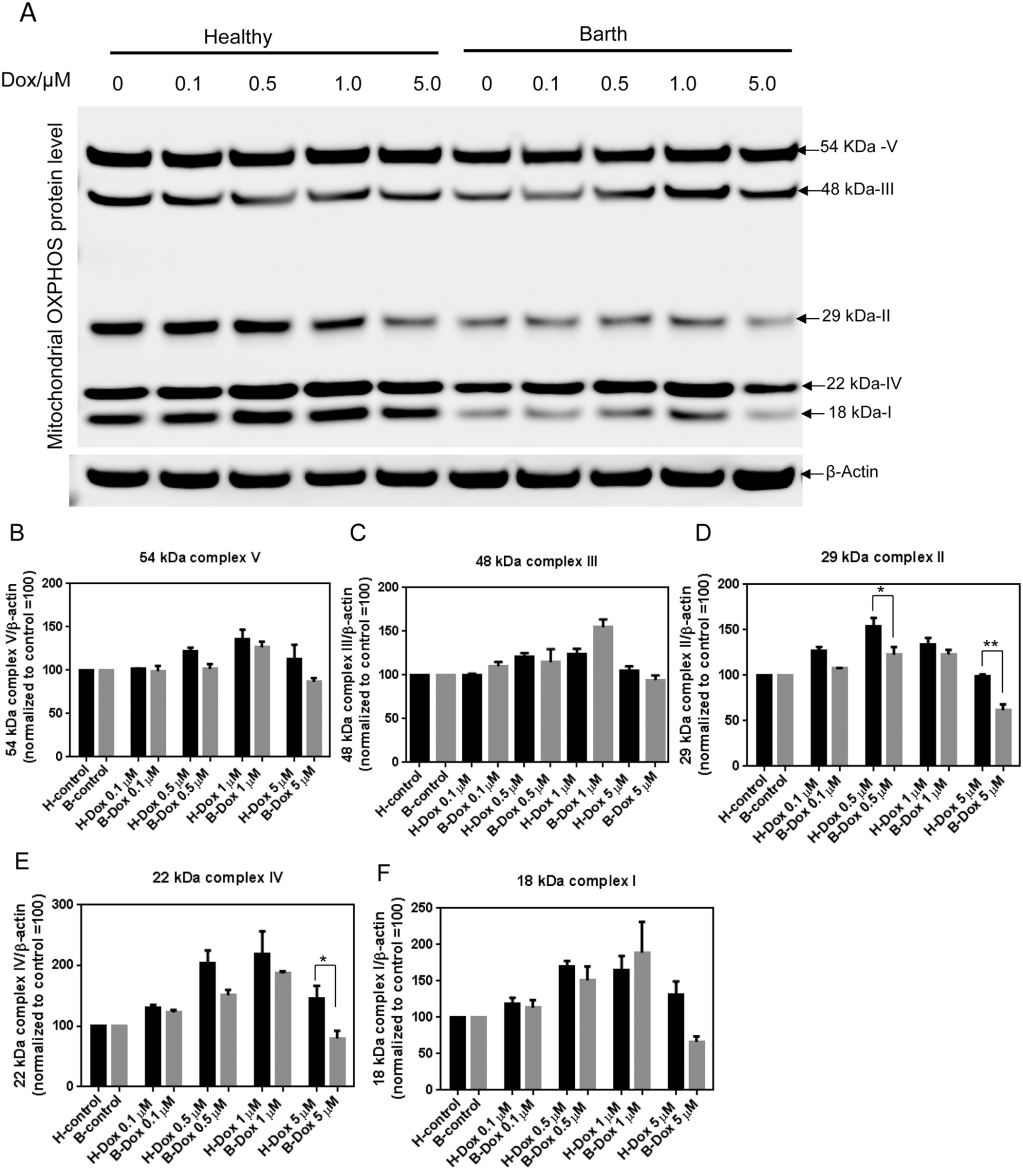
Determination of Dox-induced OXPHOS protein levels in healthy and Barth lymphocytes. (A) Representative western blot for the detection of OXPHOS proteins in healthy and Barth lymphocytes. Healthy and Barth lymphocytes were treated with 0.1, 0.5, 1, and 5 μM of Dox for 24 h and OXPHOS protein levels were determined by western blot using an antibody cocktail containing five mouse mAbs against mitochondrial complex subunits I (NDUF8-20 kDa)), II (SDHB-30 kDa), III (UQCRC2-48 kDa), IV (MTCO1-40 kDa) and V (ATP5a-55 kDa). Actin was used as a loading control to determine the relative levels of each subunit. (B-F) Quantitative determination of mitochondrial complex subunits from (A) normalized by β-actin. In each panel, basal level of mitochondrial complex subunits for control (untreated) healthy and Barth lymphocytes were normalized to 100; and Dox-induced changes in the band intensities of each subunit in the western blot were normalized against corresponding control.

The oxidative phosphorylation system (OXPHOS) is essential for cellular energy metabolism and one of the mechanisms of Dox-induced toxicity is considered to be through the alteration of OXPHOS function [35]. To evaluate the differences in OXPHOS protein levels in Barth and healthy lymphocytes following Dox treatment, we analyzed OXPHOS protein subunits by western blot. At basal conditions, OXPHOS protein levels were lower in Barth lymphocytes compared to healthy cells but Dox treatment induced a slightly increased expression of complex I, complex II, complex IV and complex V in healthy compared to Barth lymphocytes. There was no appreciable difference in Dox-induced changes in protein level of complex III between healthy and Barth lymphocytes except at 1 μM Dox treatment, which showed higher level of complex III in Barth compared to healthy lymphocytes (Fig 6A–6C). At the lower Dox concentrations tested in our experiments, we observed an increase in OXPHOS protein levels with Dox exposure. This Dox-induced increase in OXPHOS protein levels was slightly higher in healthy lymphocytes compared to Barth but at higher concentrations of Dox (5 μM), we observed that protein levels begin to decrease. This decrease was more prominent in Barth cells compared to healthy. Our data with Dox-induced enhanced OXPHOS protein complexes is in agreement with those previously shown in HCT116 colon cancer cells [36]. Dox-induced expression of complex IV was also greater in this study.

### Tafazzin knockdown in H9c2 cardiomyocytes demonstrates reduction in Dox apoptosis

**Fig 7:**
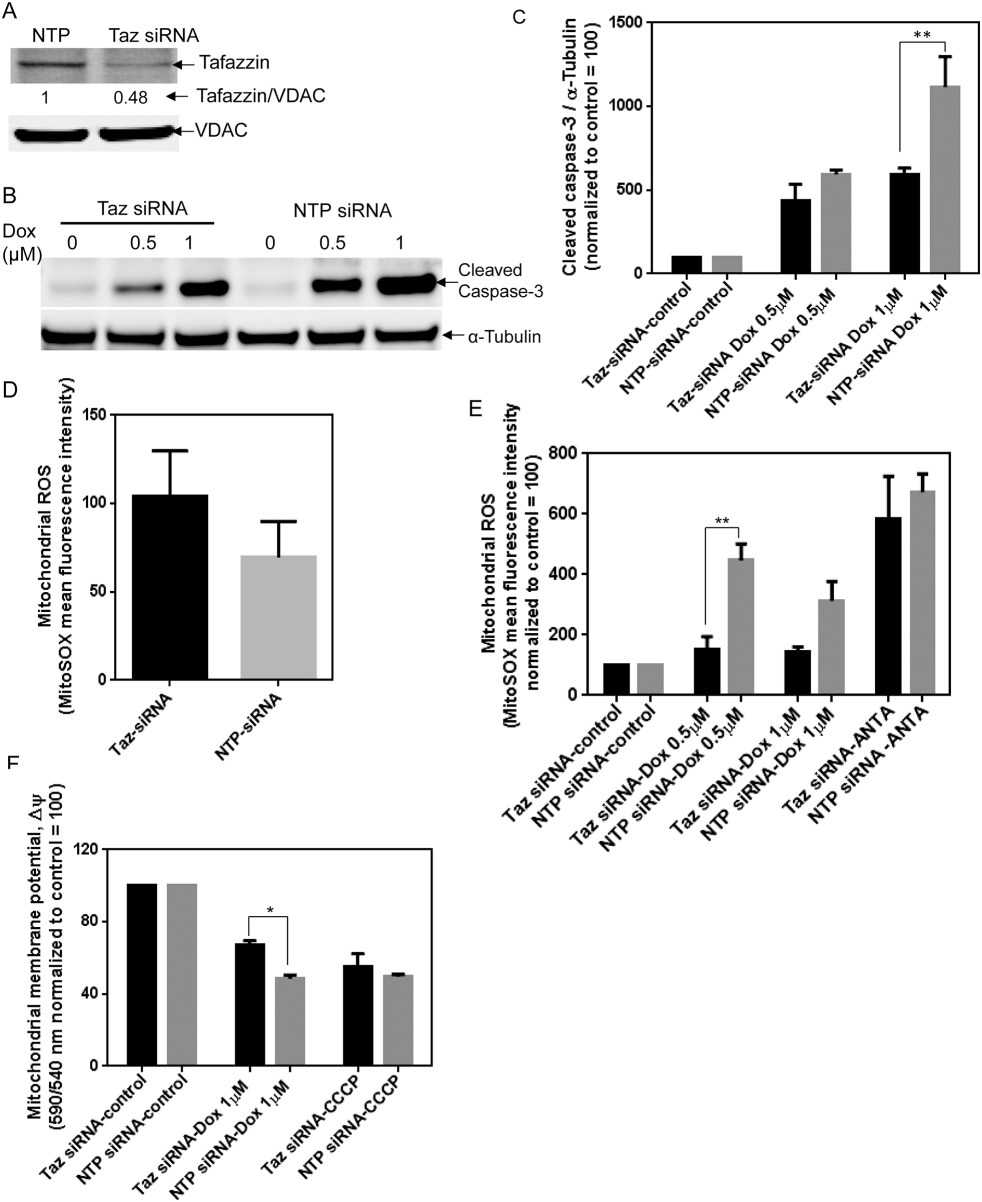
*Tafazzin* knockdown and Dox-sensitivity in H9c2 cardiomyocytes. (A) knockdown of *tafazzin* was confirmed in H9c2 cells after transfection with 20 nM *tafazzin* siRNA for 48 h by western blot analysis. (B) Representative western blot showing the level of cleaved caspase-3. After transfection with *tafazzin* and NTP siRNA for 24 h, cells were treated with 0.5 and 1 μM Dox for 24 h and cleaved caspase-3 levels were quantified using tubulin as a loading control. (C) Quantitative analysis of cleaved caspase-3 from (B) normalized by α-tubulin. (D) Mitochondrial superoxide levels were measured using MitoSOX Red dye in *tafazzin* and NTP transfected control H9c2 cells at basal levels and (E) after 0.5 and 1μM Dox treatment for 24 h. The changes in mean fluorescence intensity of mitochondrial superoxide sensitive dye MitoSOX Red in healthy and Barth lymphocytes was normalized by the corresponding control sample to compare the Dox-induced effect in both cell types. Mitochondrial complex III inhibitor Antimycin A (20 μM) was used as a positive control. (F) Measurement of mitochondrial membrane potential using JC-1 dye for NTP and Taz siRNA transfected H9c2 after treatment with Dox for 2 h. Y-axis represents the relative fluorescence ratio of 590/540 nm normalized to control without Dox treatment. Basal level of mitochondrial membrane potential in Taz siRNA transfected cells was slightly lower than NTP siRNA transfected cells (1.03 for NTP siRNA transfected cells and 0.9 for Taz siRNA transfected cells). CCCP (10 μM), a commonly used mitochondrial membrane depolarization agent was used as a positive control. To determine the Dox-induced effect between NTP and Taz siRNA transfected H9c2 cells, basal level of ROS and mitochondrial membrane potential of untreated (control) NTP and Taz siRNA transfected H9c2 cells were normalized to 100; and Dox-induced changes in the ROS and mitochondrial membrane potential were normalized against corresponding control. *p<0.05; **p<0.001.

To confirm our findings with the Barth syndrome B-cells, we investigated if a deficiency in tafazzin protein resulted in similar mitochondrial dysfunction and resulting apoptosis from Dox in a cardiac cell line. Tafazzin protein is required for the final remodeling of cardiolipin in cells [37]. The H9c2 cell line derived from embryonic rat heart tissue has been used as an *in vitro* model to study intracellular cardiac signaling and cytotoxicity. Therefore, we knocked down the *tafazzin* gene in H9c2 cells using a siRNA transfection technique to mimic CL deficient cells (Fig 7A). We assessed the cellular toxicity of Dox in *tafazzin* and NTP siRNA transfected H9c2 cells by monitoring cleaved caspase-3 levels using western blot and mitochondrial superoxide using MitoSOX Red dye. The Dox-induced cleaved caspase-3 level was higher in NTP siRNA transfected H9c2 cells compared to *tafazzin* siRNA (Taz siRNA) transfected cells (Fig 7B and 7C). The basal level of mitochondrial superoxide was higher in tafazzin deficient cells compared to normal H9c2 cells (Fig 7D). Similar to healthy B-cells, Dox-induced mitochondrial superoxide levels were higher in normal H9c2 cells compared to tafazzin deficient H9c2 cells (Fig 7E). Similarly, Taz siRNA transfected CL deficient H9c2 cells showed a reduced Dox-induced loss of mitochondrial membrane potential compared to NTP siRNA transfected H9c2 cells (Fig 7F). Taken together, these data are consistent with Dox-induced production of greater levels of superoxide and apoptosis in healthy cells compared to cardiolipin-deficient Barth cells.

## Discussion

Mitochondria are essential for cell survival, providing the majority of the energy required for cellular activities. Additionally, mitochondria also play a pivotal role in drug-induced cell death during chemotherapy. The characteristics of the mechanism by which Dox-induced ROS production leads to mitochondrial dysfunction including mitochondrial swelling, depolarization, perturbation of bioenergetics, cytochrome c release, increase in caspase-3 activity, and cell death by apoptosis are well known [38,39]. The purpose of this study was to investigate the significance of the amount of mitochondrial CL content in cells undergoing Dox-induced oxidative stress. While the binding of Dox to CL and subsequent oxidative stress-induced mitochondrial dysfunction due to redox cycling of Dox within the mitochondria is well known, there has not been a systematic study to understand the effect of mitochondrial CL content in the context of Dox-induced toxicity and whether its presence or absence directly impacts drug induced and mitochondrially-derived apoptosis [16,17]. Using CL-deficient Barth lymphocytes and tafazzin depleted H9c2 cardiomyocytes, we provide the first direct evidence that CL deficient Barth cells are better protected against Dox-induced oxidative damage.

Decreases in CL synthesis and mitochondrial CL content alter mitochondrial bioenergetics resulting in lower ATP synthesis, reduced mitochondrial membrane potential, higher mitochondrial ROS production, and higher lipid peroxidation products [6,40]. Consistent with previous finding, Barth lymphocytes showed higher mitochondrial ROS, higher lipid peroxidation products, lower mitochondrial membrane potential, and lower ATP at basal levels. However, Dox-induced oxidative damage was higher in healthy cells compared to Barth cells. Dox-induced oxidative stress is known to increase total and specific protein carbonylation which tags proteins for proteasomal degradation [41,42]. We observed higher total protein carbonylation in both cell types after Dox treatment, but level of total protein carbonylation was slightly higher in healthy cells compared to Barth cells. Using 2D gel electrophoresis we identified a specific protein, Glyceraldehyde-3-Phosphate Dehydrogenase (GAPDH) that was more carbonylated in the mitochondrial fraction of Dox treated healthy cells compared to Barth cells (S1 Fig). However, with our current data we could not conclude any mechanistic link between GAPDH carbonylation and CL-dependent Dox-induced cellular toxicity because there was no significant difference in the GAPDH biological activity between healthy and Barth cells following Dox treatment (S2 Fig). GAPDH is usually regarded as a cytosolic protein but its nuclear and mitochondrial localization has also been reported [43]. It is not clear if carbonylation of GAPDH alters its biological activity in vivo. Dox is known to increase lipid peroxidation products and malondialdehyde (MDA) is one of the common markers of lipid peroxidation products [44]. Although there was a slight increase in total protein carbonylation, we observed significantly higher Dox-induced lipid peroxidation products in healthy cells after 24 h of Dox treatment. Our data support the fact that mitochondria from healthy cells with normal levels of CL retain more Dox in the mitochondria than Barth cells, producing higher oxidative stress and mitochondrial dysfunction, leading to increased cell death. Barth cells with reduced and altered CL content retain relatively low concentrations of Dox and produce relatively lower levels of ROS and exhibit less oxidative damage compared to normal healthy cells.

ATP levels were decreased after Dox treatment but OXPHOS protein levels were increased with lower concentrations of Dox, and then decreased with a higher Dox concentration (5 μM). At basal conditions, OXPHOS protein levels were lower in Barth cells compared to healthy, indicating an impaired OXPHOS system due to the lower CL concentration in Barth cells. However, after Dox treatment, OXOPHOS protein levels were slightly higher in healthy cells compared to Barth cells with the exception of complex III. The Dox-induced increase in TCA cycle activity and respiration accompanied by diminished ATP levels has been shown previously and is in agreement with our findings [36,45]. Enhanced mitochondrial respiration and higher OXPHOS levels following Dox treatment has been attributed to a compensatory effect where cells try to compensate a decrease in ATP levels during apoptosis by increasing respiration and OXPHOS activity.

Mitochondrial and CL content is much higher in cardiac muscle compared to skeletal and smooth muscle [46,47]. Therefore, we used H9c2 cardiomyocyte cells to further understand the involvement of CL in Dox-induced toxicity. Previous studies have shown that the knockdown of *tafazzin* in cardiac myocytes decreases cardiolipin content and increases mitochondrial ROS production [40]. Consistent with previous findings and our results in Barth lymphocytes, knockdown of *tafazzin* in H9c2 cells showed higher basal levels of mitochondrial ROS without Dox treatment but the Dox-induced mitochondrial ROS was lower in tafazzin deficient H9c2 cells. Overall, our data support that Dox-CL interaction and mitochondrial accumulation of Dox is the major cause of Dox-induced oxidative damage. Depletion of tafazzin reduces mitochondrial CL and hence mitochondrial Dox content, thereby reducing the Dox-induced mitochondrial damage.

A recent study shows that, SS-31, a cardiolipin protective mitochondrially-targeted peptide, reduces Dox-induced mitochondrial ROS and protects mitochondria against Dox-induced toxicity [48]. SS-31 binds with cardiolipin within inner mitochondrial membrane and inhibits peroxidase activity of cytochrome c while maintaining its electron carrying function [14]. This protective effect has been attributed to selective higher affinity of SS-31 peptide for CL and consequently preventing Dox binding to CL, thus preventing oxidative damage to mitochondria [49].

CL is important to maintain proper mitochondrial function. Oxidation of CL, decrease in CL content, and alteration of CL composition affect mitochondrial function [50]. A decrease in CL has been shown under various pathological conditions and with aging. Since CL plays a crucial role in Dox-induced toxicity, patients with low CL content or altered composition may be less susceptible to the off-target effects of Dox. Thus, development of therapeutic agents that selectively bind to CL and inhibit Dox-CL interaction without affecting the integrity of ETC might have a therapeutic advantage to resist Dox-induced toxicity.

## Supporting Information

S1 Fig Identification of specific carbonylated proteins by 2D gel electrophoresis. Representative 2D gel and western blot for healthy and Barth lymphocytes with and without Dox treatment. The small box and arrow in each figure panel represents the specific protein that was more carbonylated in healthy cells compared to Barth after Dox treatment.
(TIF)

S2 Fig Measurement of GAPDH activity in healthy and Barth lymphocytes. Quantitative data representing the GAPDH activity in healthy and Barth lymphocytes with and without Dox treatment. IAA = iodoacetamide
(TIF)

S1 File Experimental details of 2D gel electrophoresis, protein identification, and GAPDH activity. (DOC)
