# Supplementary figures and images for: Deficiency in Cardiolipin Reduces Doxorubicin-Induced Oxidative Stress and Mitochondrial Damage in Human B-Lymphocytes
Source: PLoS One. 2016 Jul 19;11(7):e0158376. doi: 10.1371/journal.pone.0158376 (PMC4951097; doi:10.1371/journal.pone.0158376)

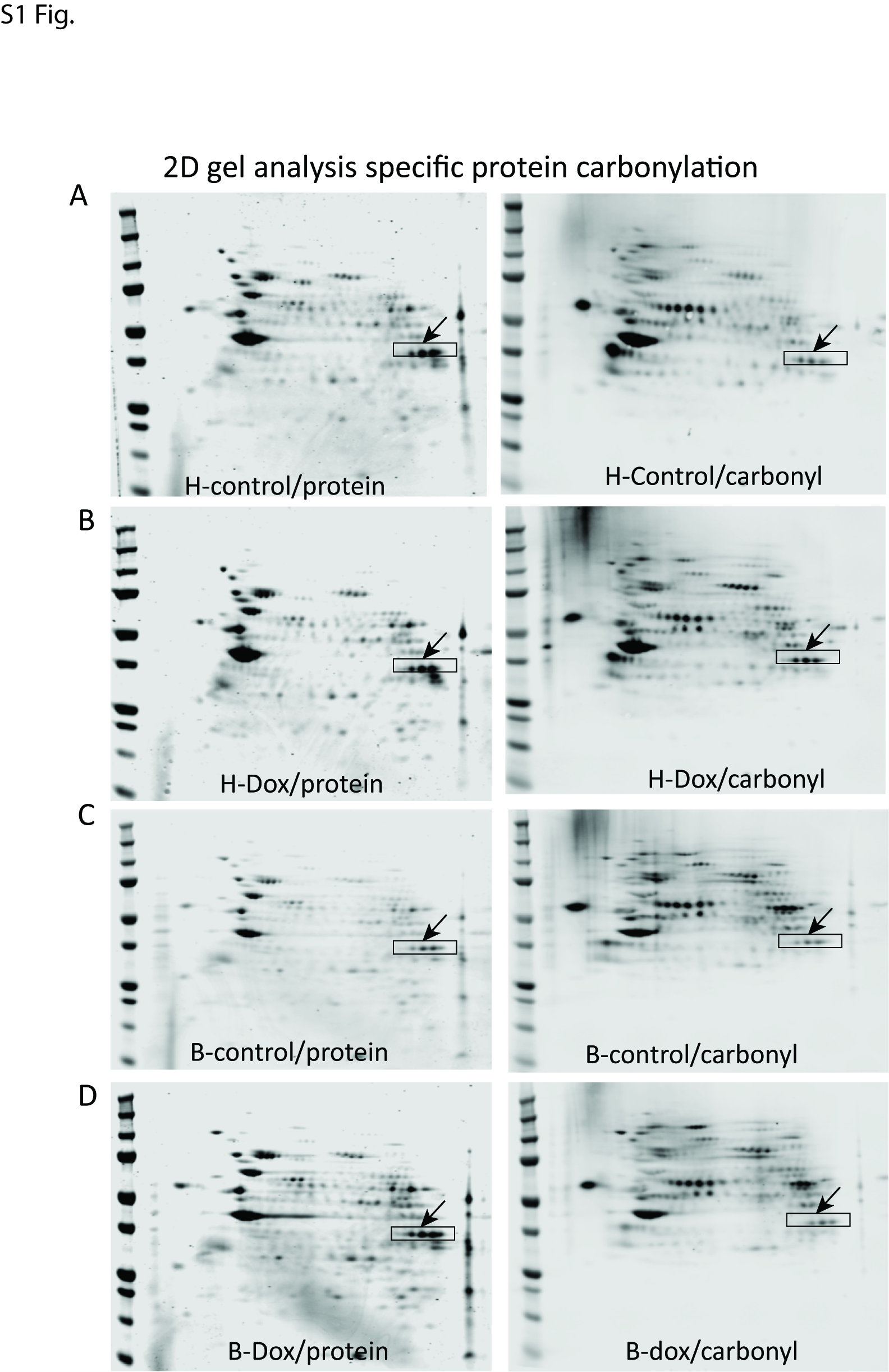

Supplement: S1 Fig — Representative 2D gel and western blot for healthy and Barth lymphocytes with and without Dox treatment. The small box and arrow in each figure panel represents the specific protein that was more carbonylated in healthy cells compared to Barth after Dox treatment. (TIF) [file pone.0158376.s001.tif]

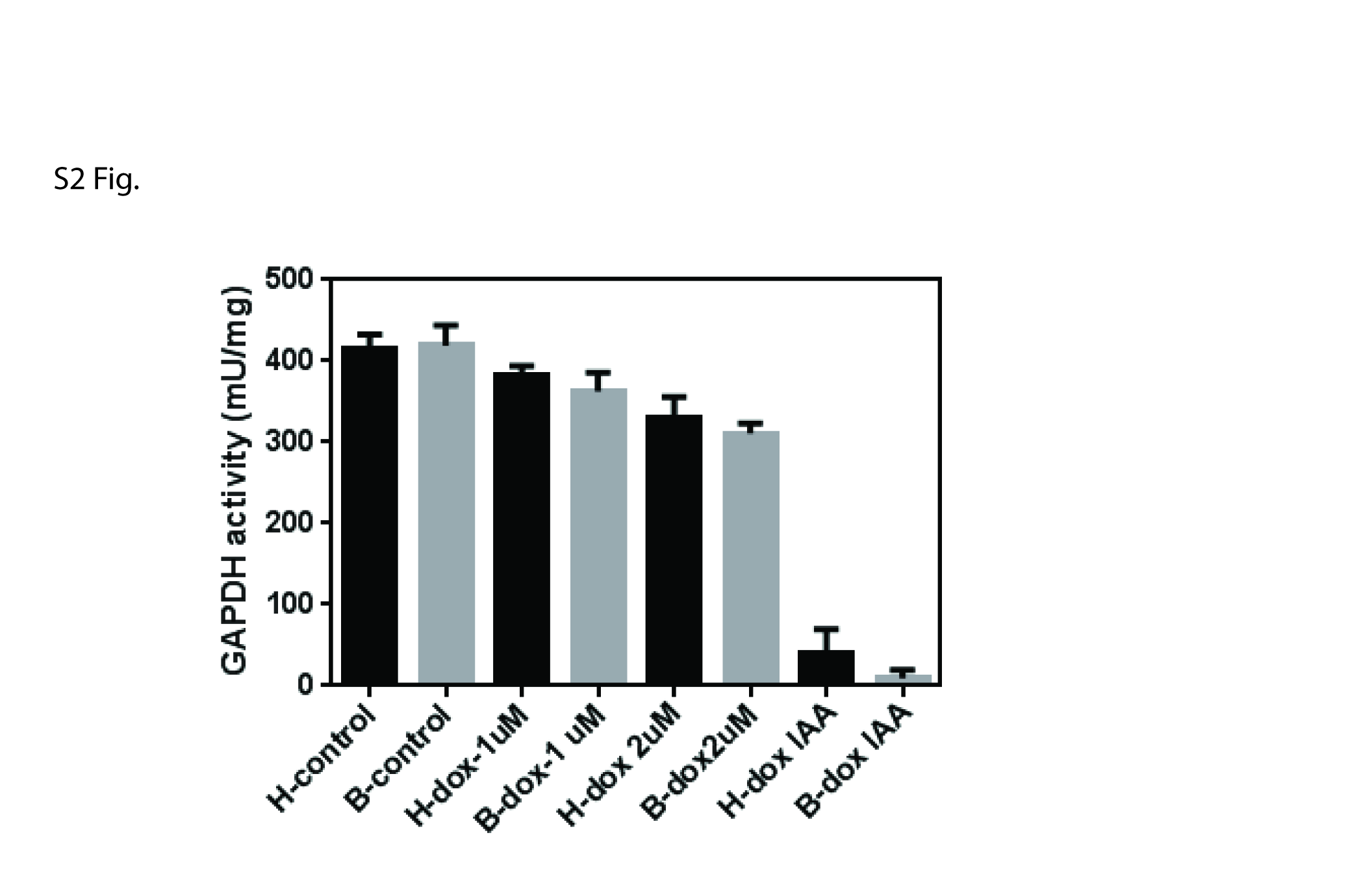

Supplement: S2 Fig — Quantitative data representing the GAPDH activity in healthy and Barth lymphocytes with and without Dox treatment. IAA = iodoacetamide (TIF) [file pone.0158376.s002.tif]
