# Supplementary material for: Deficiency in Cardiolipin Reduces Doxorubicin-Induced Oxidative Stress and Mitochondrial Damage in Human B-Lymphocytes
Source: PLoS One. 2016 Jul 19;11(7):e0158376. doi: 10.1371/journal.pone.0158376 (PMC4951097; doi:10.1371/journal.pone.0158376)
Supplement: S1 File — (DOC) [file pone.0158376.s003.doc]

**S1 File. Experimental details of 2D gel electrophoresis, protein identification and GAPDH activity.**

*2D gel electrophoresis*

Cells were treated with 1 µM Dox or DMSO for 24 h and mitochondrial fraction was isolated from cells. Mitochondrial fractions were denatured in 6% SDS and treated with 20 mM DNPH in 10% TFA to a final concentration of 10 mM DNPH and incubated for 10 minutes at room temperature. Proteins were precipitated with trichloroacetic acid (TCA) to a final concentration of 20% for 15 minutes on ice. Following centrifugation, precipitated proteins were washed with an ethanol/ethyl acetate mixture (1:1 v/v) three times followed by a final wash with pre-chilled 90% acetone in water. The precipitates were allowed to air dry and dissolved in rehydration buffer composed of 7 M urea, 2 M thiourea, 2% CHAPS, 0.5% ampholytes pH 3–10, and 0.002% (w/v) bromophenol blue and 50 mM DTT. Then, 40 µg of each sample was applied to a 7 cm IPG strip and rehydrated overnight at room temperature. The rehydrated IPG strips containing samples were isoelectrically focused on an IPGphor (General Electric, Pittsburgh, PA). After isoelectric focusing, IPG strips were equilibrated with equilibration buffer I (2% SDS, 50 mM Tris- HCl pH 8.8, 6M urea, 30% (v/v) glycerol, 0.002% bromophenol blue, and 1% DTT) followed by equilibration buffer II (2% SDS, 50 mM Tris-HCl pH 8.8, 6M urea, 30%(v/v) glycerol, 0.002% bromophenol blue, and 2.5% iodoacetamide) for 15 minutes each. Two-dimensional gel electrophoresis was performed on the strips to separate proteins according to their molecular weights. Following transfer to PVDF membrane, the membrane was blocked and treated with goat anti-DNP primary antibody (Bethyl Laboratories Inc., Montgomery, TX) followed by donkey anti-goat IRDye 800CW secondary antibody (LI-COR, Lincoln, NE). DNP-derivatized carbonylated proteins were detected using the Odyssey infrared imaging system (LI-COR, Lincoln, NE). Densitometric intensity of the major carbonylated protein spots in the 2D-Western western blots were divided by the corresponding protein spots in the Coomassie blue staining gel to determine the carbonylation of corresponding proteins.

*Protein identification*

Drug-induced carbonylated proteins were compared in Western western blot from healthy and Barth lymphocytes, gel spots corresponding to highly carbonylated proteins were excised from the gel and digested with sequencing grade modified trypsin (Promega Corporation, Madison, WI). Trypsin digested peptides were subjected to LC-MS/MS analysis using the Q-TOF LC/MS (Agilent Technologies, Santa Clara, CA). Peptide search and protein identification was performed using the Spectrum Mill MS Proteomic Workbench (Agilent Technologies, Santa Clara, CA).

*GAPDH activity*

GAPDH activity was measured in the cell lysates using the GAPDH activity assay kit (BioVision, Milpitas, CA) according to the manufacturer’s protocol. Briefly, healthy and Barth B-lymphocytes were grown in 6-well plates and treated with Dox for 24 h or 0.3 mM iodoacetamide (IAA) for 1 h . About 1x10^6^ cells per treatment were lysed in GAPDH assay buffer and GAPDH activity was measured in a 96-well plate at 540 nm using the SpectraMax i3 plate reader (Molecular Devices, Sunnyvale, CA). GAPDH activity was normalized by protein concentration and expressed as mU/mg. IAA was used a positive control to inhibit GAPDH activity.
